# Vibration Analysis of Composite Laminate Plate Excited by Piezoelectric Actuators

**DOI:** 10.3390/s130302997

**Published:** 2013-03-01

**Authors:** Shiuh-Chuan Her, Chi-Sheng Lin

**Affiliations:** Department of Mechanical Engineering, Yuan Ze University, Chung-Li 320, Taiwan; E-Mail: s917224@mail.yzu.edu.tw

**Keywords:** piezoelectric actuator, composite laminate, smart structure, harmonic vibration

## Abstract

Piezoelectric materials can be used as actuators for the active vibration control of smart structural systems. In this work, piezoelectric patches are surface bonded to a composite laminate plate and used as vibration actuators. A static analysis based on the piezoelectricity and elasticity is conducted to evaluate the loads induced by the piezoelectric actuators to the host structure. The loads are then employed to develop the vibration response of a simply supported laminate rectangular plate excited by piezoelectric patches subjected to time harmonic voltages. An analytical solution of the vibration response of a simply supported laminate rectangular plate under time harmonic electrical loading is obtained and compared with finite element results to validate the present approach. The effects of location and exciting frequency of piezoelectric actuators on the vibration response of the laminate plate are investigated through a parametric study. Numerical results show that modes can be selectively excited, leading to structural vibration control.

## 1. Introduction

Smart structures incorporating active devices to sense and actuate the structure could be applied in many engineering applications, such as aircraft structures, satellites, large space structures and so forth [1,2]. Many materials have been tested as actuators and sensors such as piezoelectric materials, shape memory alloys, electrostrictive materials, magnetostrictive materials, electro-rheological fluids, and fiber optics [3]. These materials can be embedded into or surface bonded with structures, thus acting as either a sensor or an actuator. Among them, piezoelectric materials have become popular because of high strength, temperature insensitivity, and ease of implementation. Active vibration control of flexible structures using piezoelectric sensors and actuators has received much attention in recent years. Successful implementation of active adaptive control is usually based on the full understanding of the smart structure system, which significantly affects the control strategy, parameter tunings and convergence [4]. This process has proved to be tedious and difficult if it is done only by experiments. Thus, suitable simulation models are necessary to facilitate the development. Numerous efforts have been made in the last few years to develop suitable simulation models. Alibeigloo and Madoliat [5] presented a three-dimensional solution for static analysis of a cross-ply rectangular plate imbedded in piezoelectric layers using the differential quadrature method (DQM) and a Fourier series approach. Proulx and Cheng [6] reported a dynamic model for the active vibration control of a rectangular plate using piezoceramic elements of arbitrary shape. Susanto [7] presented an analytical model of piezoelectric laminated slightly curved beams, which included the computation of natural frequencies, mode shapes and transfer function formulation using the distributed transfer function method. Qiu *et al.* [8] used piezoelectric ceramic patches as sensors and actuators to suppress the vibration of a smart flexible clamped plate. An optimal placement method for the locations of piezoelectric actuators and sensors was developed based on the degree of observability and controllability indices for the cantilever plate. Santos *et al.* [9] developed a finite element model for the analysis of 3D axisymmetric laminated shells with piezoelectric sensors and actuators. Fernandes and Pouget [10] predicted the static and dynamic (vibration) responses of composite plates excited by piezoelectric actuators based on the kinematic assumption of the Love-Kirchhoff thin plate theory. The influence of the actuator position on the global and local responses of the composite plate was illustrated. Qing *et al.* [11] employed a modified mixed variational principle for piezoelectric materials to establish a semi-analytical solution for static and dynamic analysis of plates with piezoelectric patches. Huang and Sun [12] studied the load transfer and wave propagation of an anisotropic elastic medium induced by the surface bonded piezoelectric actuator. Dimitriadis *et al.* [13] used two dimensional patches of piezoelectric material bonded to the surface of a simply supported plate as vibration actuators to excite the selected modes. Qu *et al.* [14] proposed a dynamical model using the principle of minimum energy to study the vibration behavior of a piezoelectric composite plate with cracks, the effects of cracks and piezoelectric materials on mode shapes was presented. Della and Shu [15] presented a mathematical model basing on the Euler-Bernoulli beam theory and Rayleigh-Ritz approximation technique for the vibration of beams with embedded arrays of piezoelectric sensors and actuators. Smart structures often involve a coupling model between the host structure and piezoelectric sensors and actuators. In fact, a piezoelectric actuator introduces material and geometric discontinuities which lead to some mathematical difficulties. Because of the difficulties associated with the complicated electromechanical coupling, material inhomogeneity and anisotropy, analytical solutions representing the vibration behavior of a composite plate excited by PZT actuators have not been properly established. Most of the existing works focused on the numerical solutions such as finite element method. In this study, a theoretical model accomplished with analytical solution is presented to show how patch type piezoelectric actuators can be used to excite and control the vibration modes of a composite laminate plate.

The present work investigated the vibration response of a simply supported composite laminate plate excited by piezoelectric actuators. The model consists of two piezoelectric patches symmetrically surface bonded on a cross-ply composite laminate. Static analysis leads to the determination of the loads induced by the piezoelectric actuators to the host structure. This is followed by a dynamic analysis for a composite laminate plate excited by piezoelectric actuators with time harmonic electrical loading. A closed form solution to the harmonic vibration of the simply supported composite laminate was obtained by using the classical laminate plate theory. The analytical solution was validated by the corresponding finite element results. Results are presented for the vibration displacement distribution and modal shape of the composite laminate plate. The effects of excitation frequency and actuator location on the vibration displacement and modal shape are investigated through a parametric study.

## 2. Bending Moment

In this work, two piezoelectric actuators are symmetrically bonded on the top and bottom surfaces of a cross-ply composite laminate. The polarized direction is along the z-axis. For an unconstrained thin piezoelectric actuator, equal strains in both x and y directions will be induced when activated by a voltage along the poling direction. The magnitude of the strain can be expressed in terms of the piezoelectric constant *d*_31_, applied voltage *V* and actuator thickness *t_pe_*, as follows:

(1) $${\left({ɛ}_{x}\right)}_{pe}={\left({ɛ}_{y}\right)}_{pe}={ɛ}_{pe}=\frac{d_{31}}{t_{pe}}V$$

where subscripts *pe* and *p* represent the quantities associated with piezoelectric actuator and host plate, respectively, throughout this paper. When an electrical field is applied in the direction normal to the actuator surface, surface strains are generated Equation (1). Due to the coupling of the actuator to the structure, forces and moments are induced in the bonded area of the structure. Since this work focuses on the deformation of the plate induced by the bending moment, only constant *d_31_* is considered in this model.

**Figure 1.**
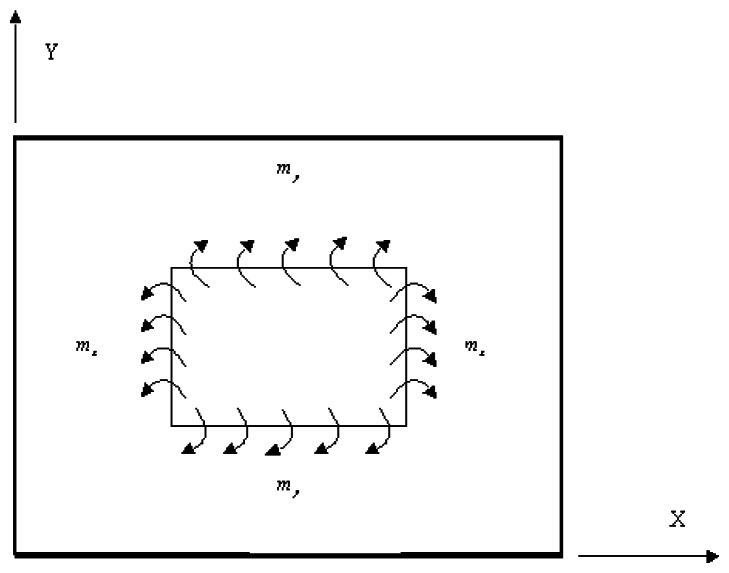
Bending moment acting on the composite laminate induced by piezoelectric actuators.

The two actuators are activated by applying a voltage of equal magnitude and opposite sign to the opposing actuators. The opposite directions of the surface tractions at the interfaces between the actuator and plate cause the uniform bending moments along the actuator boundaries as shown in Figure 1.

The bending moments per unit length *m_x_* and *m_y_* induced by the actuators to the cross-ply composite laminate have been derived in the previous work [16] as follows:

(2a) $$m_{x}=C_{1}{ɛ}_{pe}$$

(2b) $$m_{y}=C_{2}{ɛ}_{pe}$$

(3a) $${\text{C}}_{1}=({\text{A}}_{1}{\left({\text{D}}_{11}\right)}_{\text{p}}+{\text{A}}_{2}{\left({\text{D}}_{12}\right)}_{\text{p}})$$

(3b) $${\text{C}}_{2}=({\text{A}}_{1}{\left({\text{D}}_{12}\right)}_{\text{p}}+{\text{A}}_{2}{\left({\text{D}}_{12}\right)}_{\text{p}})$$

(4a) $$A_{1}=\frac{B_{11pe}(1+v_{pe})}{{\left(D_{11}\right)}_{p}+D_{11pe}}+\frac{\left(({\left(D_{12}\right)}_{p}+D_{12pe})(-B_{11pe}({\left(D_{11}\right)}_{p}+D_{11pe})(1+v_{pe})+B_{11pe}({\left(D_{12}\right)}_{p}+D_{12pe})(1+v_{pe}))\right)}{\left(({\left(D_{11}\right)}_{p}+D_{11pe})(-{\left({\left(D_{12}\right)}_{p}+D_{12pe}\right)}^{2}+({\left(D_{11}\right)}_{p}+D_{11pe})({\left(D_{22}\right)}_{p}+D_{22pe}))\right)}$$

(4b) $$A_{2}=\frac{-B_{11Pe}({\left(D_{11}\right)}_{p}+D_{11pe})(1+v_{pe})+B_{11pe}({\left(D_{12}\right)}_{p}+D_{12pe})(1+v_{pe})}{-{\left({\left(D_{12}\right)}_{p}+D_{12pe}\right)}^{2}+({\left(D_{11}\right)}_{p}+D_{11pe})({\left(D_{22}\right)}_{p}+D_{22pe})}$$

Where *B_11_*, *D_1_*, *D_12_*, *D_22_* are related to the stiffness of the piezoelectric material and composite material.

## 3. Harmonic Vibration of a Simply Supported Composite Plate

**Figure 2.**
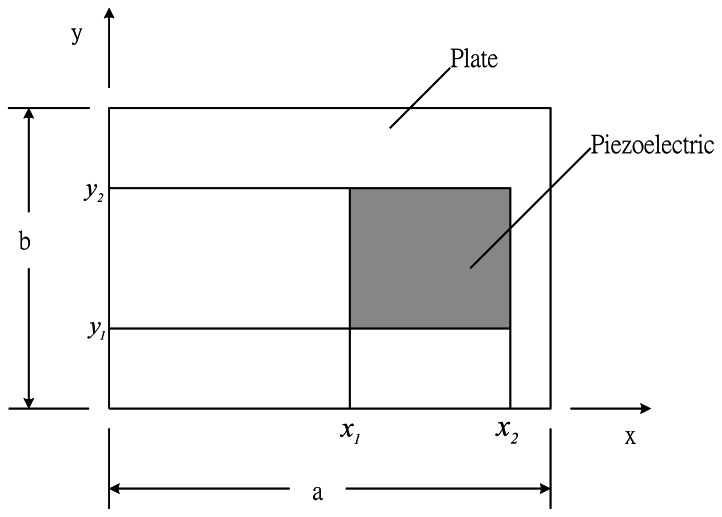
Surface bonded actuator on the composite laminate plate.

The host plate is a rectangular plate with simply supported boundary conditions. The location of the surface bonded actuators viewed from the top is shown in Figure 2. The activated piezoelectric actuators will induce bending moments as shown in Equation (2) to the composite plate and can be expressed in terms of unit step functions as follow:

(5a) $$m_{x}=C_{1}{ɛ}_{pe}[h(x-x_{1})-h(x-x_{2})][h(y-y_{1})-h(y-y_{2})]$$

(5b) $$m_{y}=C_{2}{ɛ}_{pe}[h(x-x_{1})-h(x-x_{2})][h(y-y_{1})-h(y-y_{2})]$$

If the actuator input voltage is sinusoidal oscillating with frequency *p*, the bending moments *m_x_* and *m_y_* will oscillate at the same frequency as the voltage. Using the classical laminate plate theory, the equation of motion for the plate can be written in terms of the displacement *w* and the actuators induced moments *m_x_* and *m_y_* as:

(6) $$\begin{array}{c} {\left(D_{11}\right)}_{p}\frac{\partial^{4}w}{\partial x^{4}}+2H_{1}\frac{\partial^{4}w}{\partial x^{2}\partial y^{2}}+{\left(D_{22}\right)}_{p}\frac{\partial^{4}w}{\partial y^{4}}+m^{\prime \prime }\ddot{w}=F(x,y)\sin pt\\ H_{1}={\left(D_{12}\right)}_{p}+2{\left(D_{66}\right)}_{p}\\ F(x,y)=\frac{\partial^{2}m_{x}}{\partial x^{2}}+\frac{\partial^{2}m_{y}}{\partial y^{2}}=C_{1}({ɛ}_{pe})[\delta^{\prime }(x-x_{1})-\delta^{\prime }(x-x_{2})][h(y-y_{1})-h(y-y_{2})]+C_{2}({ɛ}_{pe})[h(x-x_{1})-h(x-x_{2})][\delta^{\prime }(y-y_{1})-\delta^{\prime }(y-y_{2})] \end{array}$$

Where (D_11_)*_p_*, (D_22_)*_p_*, (D_66_)*_p_* are the bending stiffness of the composite laminate, *m″* is the area mass density of the composite plate.

The forcing function *F(x, y)* can be expressed in terms of a Fourier series as follows:

(7) $$\begin{array}{c} F(x,y)=(\sum_{m=1}^{\infty }\sum_{n=1}^{\infty }F_{mn}\sin\frac{m\pi x}{a}\sin\frac{n\pi y}{b})\\ F_{mn}=\frac{4}{a*b}\int_{0}^{b}\int_{0}^{a}F(x,y)\sin\frac{m\pi x}{a}\sin\frac{n\pi y}{b}dxdy=\frac{4}{a*b}[-\frac{C_{2}{ɛ}_{pe}\gamma_{m}^{2}+C_{1}{ɛ}_{pe}\gamma_{n}^{2}}{\gamma_{m}\gamma_{n}}(\cos\gamma_{m}x_{1}-\cos\gamma_{m}x_{2})(\cos\gamma_{n}y_{1}-\cos\gamma_{n}y_{2})]\\ \gamma_{m}=\frac{m\pi }{a};\gamma_{n}=\frac{n\pi }{b} \end{array}$$

For a simply supported rectangular plate, the flexural displacement *w* can be expressed by the following Fourier series:

(8) $$w(x,y,t)=\sum_{m=1}^{\infty }\sum_{n=1}^{\infty }W_{mn}\sin\frac{m\pi x}{a}\sin\frac{n\pi y}{b}\sin pt$$

Substituting Equations (7) and (8) into Equation (6), yields:

(9) $$W_{mn}(D_{11}\frac{m^{4}\pi^{4}}{a^{4}}+2H_{1}\frac{m^{2}\pi^{2}}{a^{2}}\frac{n^{2}\pi^{2}}{b^{2}}+D_{22}\frac{n^{4}\pi^{4}}{b^{4}}-m^{\prime \prime }p^{2})=F_{mn}$$

The natural frequency of a simply supported composite plate is:

(10) $$\omega_{mn}=\pi^{2}\sqrt{\frac{D_{11}\frac{m^{4}}{a^{4}}+2H_{1}\frac{m^{2}n^{2}}{a^{2}b^{2}}+D_{22}\frac{n^{4}}{b^{4}}}{m^{\prime \prime }}}$$

Substituting Equation (10) into Equation (9), results:

(11) $$W_{mn}=\frac{F_{mn}}{m^{\prime \prime }(\omega_{mn}^{2}-p^{2})}$$

Thus, the vibration displacement of the composite plate can be obtained by substituting Equation (11) into Equation (8) as follows:

(12) $$w(x,y,t)=[\sum_{m=1}^{\infty }\sum_{n=1}^{\infty }\frac{F_{mn}}{m^{\prime \prime }(\omega_{mn}^{2}-p^{2})}\sin\frac{m\pi x}{a}\sin\frac{n\pi y}{b}]\sin pt$$

## 4. Finite Element Analysis

The finite element method is a widely used and powerful tool for analyzing complex structures. Many researchers have modelled the piezoelectric actuation using the finite element method. The commercially available finite element software ANSYS has the ability to analyze piezoelectric materials. In this study, ANSYS is adopted to investigate the harmonic vibration of a simply supported composite plate excited by the surface bonded piezoelectric actuators. To perform the ANSYS finite element analysis for the piezoelectric actuator bonded structure, SOLID 45 and SOLID 5 elements were used in the composite plate and piezoelectric actuators, respectively.

**Figure 3.**
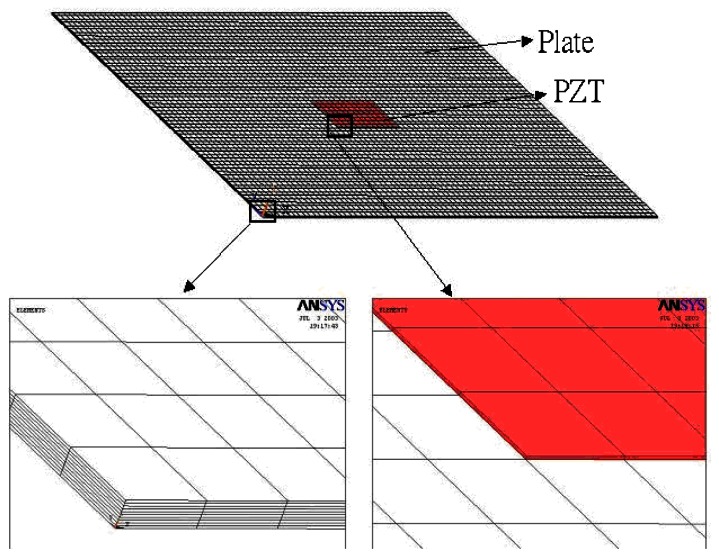
3-D finite element mesh.

A typical three dimensional finite element mesh is shown in Figure 3. A time harmonic voltage between the upper and lower surfaces of the SOLID 5 elements is applied, which results in an electric field along the poling direction of the actuator. The vibration displacements obtained from the finite element method are compared with the analytical solutions of Equation (12) to validate the present approach.

## 5. Parametric Study and Verification

In the following numerical examples, the composite material is carbon/epoxy with stacking sequence [0/90/90/0].The composite material properties of the carbon/epoxy are listed in Table 1. The dimensions of the composite laminate plate are length *a* = 0.38 m, width *b* = 0.3 m, thickness *t_p_* = 1.5876 mm. The piezoelectric actuator is assumed to be a PZT G-1195 with the following material properties [17]: Young's modulus *E_pe_* = 63 GPa, Poisson's ratio *v_pe_* = 0.3, density *ρ_pe_* = 7,600 kg·m^−2^, piezoelectric constant *d_31_* = 1.9 × 10^−10^ V·m^−1^ and thickness *t_pe_* = 0.15876 mm. The effects of the excitation frequency and location of the actuators are presented through a parametric study to investigate the vibration shape of the composite plate activated by the surface bonded piezoelectric actuators.

**Figure 4.**
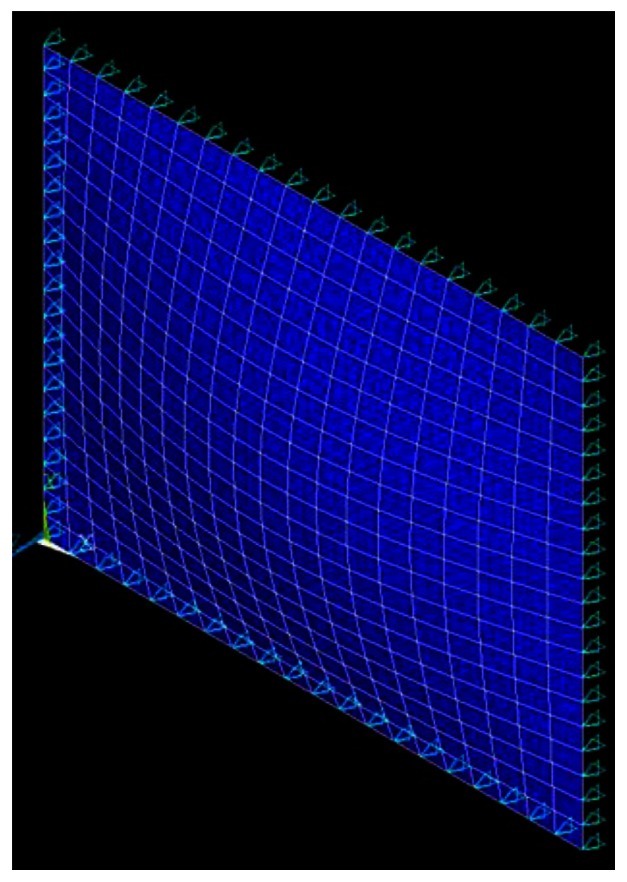
Mode shape of (1,1).

**Figure 5.**
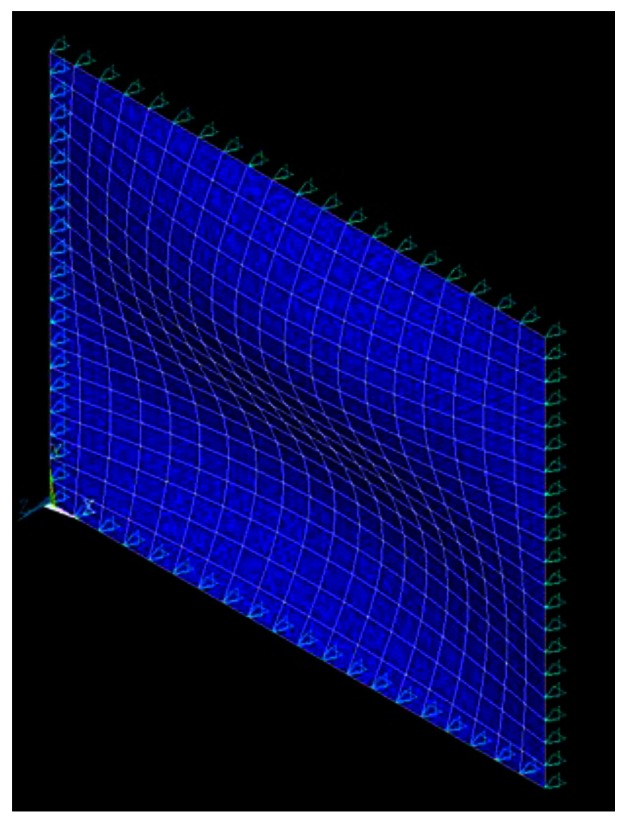
Mode shape of (1,2).

**Figure 6.**
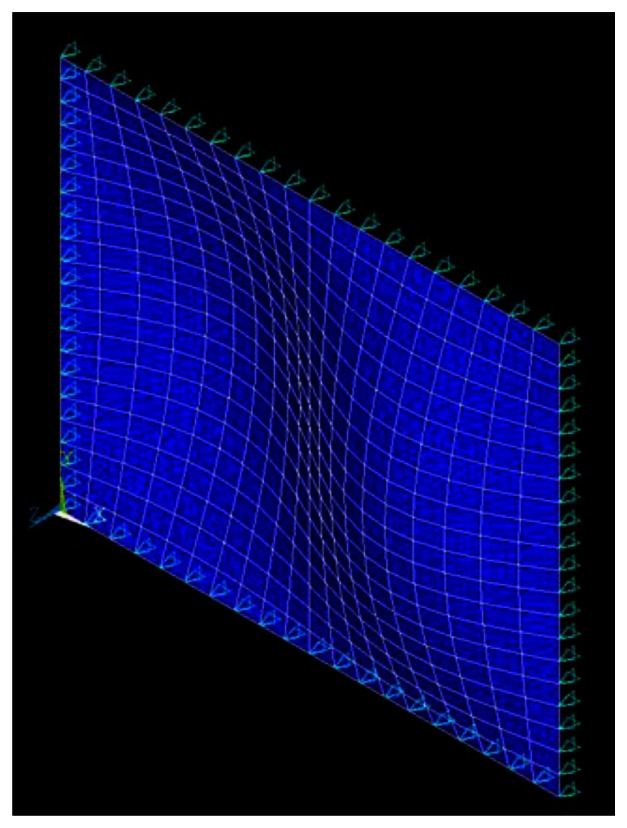
Mode shape of (2,1).

**Figure 7.**
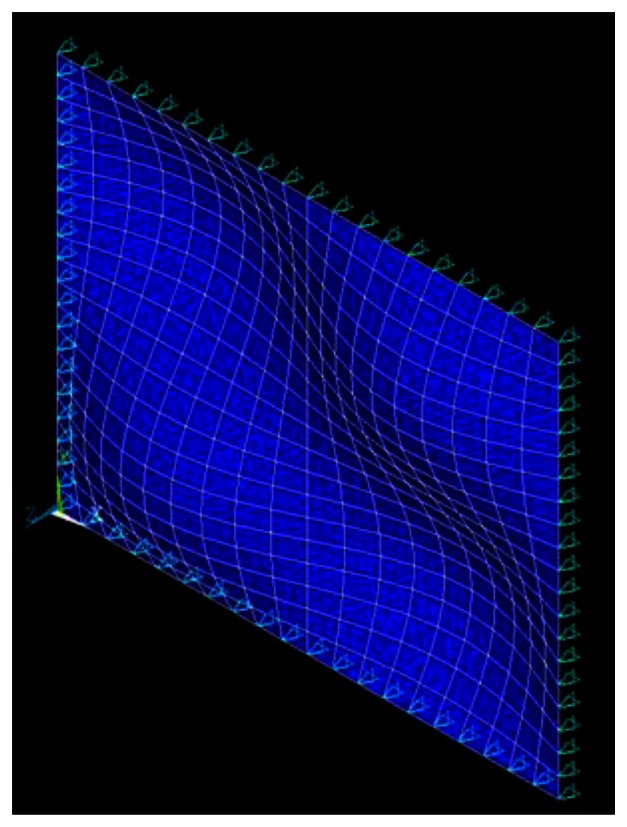
Mode shape of (2,2).

The natural frequencies of the simply supported composite laminate plate obtained by the finite element method and theoretical prediction Equation (10) are listed in Tables 2 and 3, respectively. It shows that the theoretical predictions agree well with the finite element results. The mode shapes of modes (1,1), (1,2), (2,1) and (2,2) are shown in Figures 4, 5, 6, and 7, respectively.

Example results are presented for the harmonic vibration of a rectangular composite thin plate excited by a pair of rectangular piezoelectric actuators with various dimensions and locations. To excite the selective vibration mode, the excitation frequency can be determined using Equation (10). Four different excitation frequencies (350, 650, 870 and 1,050 rads/s) were tested. Among these excitation frequencies, 350, 870 and 1,050 rads/s are close to the resonant frequencies of the (1,1), (1,2) and (2,1) modes, respectively, while the 650 rads/s is far away from those resonant frequencies. The vibration displacements calculated by the theoretical prediction (Equation (12)) are compared with the finite element results. In this work, the forcing function F(x, y,) as shown in Equation (7) was expressed in terms of Fourier series. Convergence test results show that 10 terms of Fourier series can converge to a correct solution. Thus, 10 terms of Fourier series was adopted throughout this analysis.

### 5.1. Three Different Sizes of Piezoelectric Actuators

**Figure 8.**
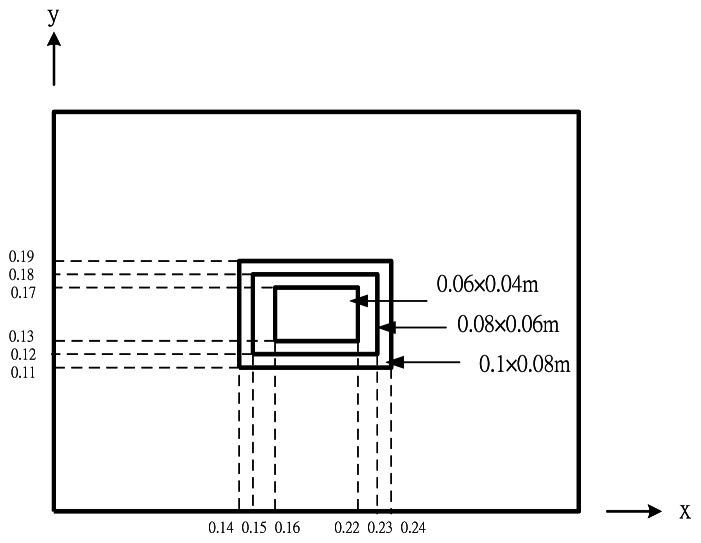
Three different sizes of PZT actuators.

Two piezoelectric actuators are surface bonded on the top and bottom surfaces of the composite plate. Three different sizes of piezoelectric actuators with the dimensions of 0.06 m × 0.04 m, 0.08 m × 0.06 m and 0.1 m × 0.08 m, respectively, bonded on the central area of the composite plate as shown in Figure 8 are considered in this example.

The piezoelectric actuators are excited by time harmonic voltages with three different frequencies of 350, 650 and 870 rads/s, respectively.

#### 5.1.1. Excitation Frequency *p* = *350 rads/s*

**Figure 9.**
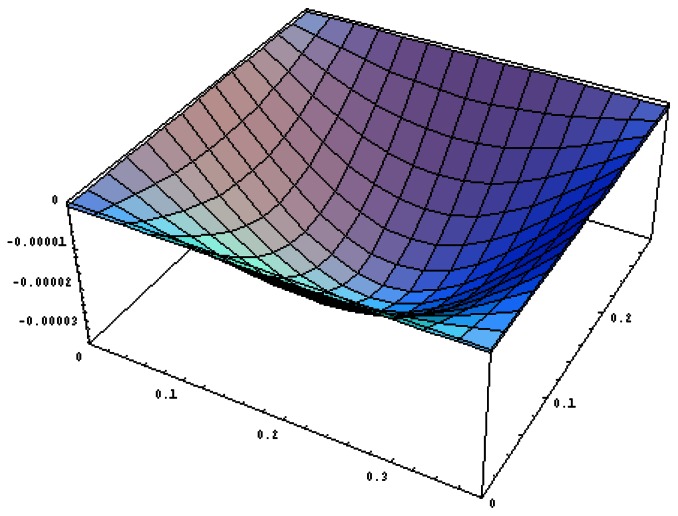
Vibration profile of the composite plate excited by PZT actuator bonded on the center of the plate with excitation frequency 350 rads/s.

**Figure 10.**
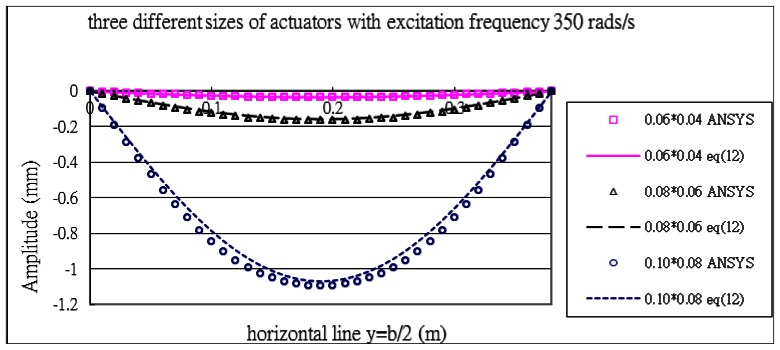
Vibration displacements of the composite plate obtained by ANSYS and Equation (12) along the horizontal line (*y* = *b*/2) excited by PZT actuator bonded on the center of the plate with excitation frequency 350 rads/s for three different sizes of actuators.

Figure 9 shows the vibration profile of the composite plate excited by the PZT actuators with excitation frequency of 350 rads/s. As can be seen from Tables 2 and 3, this frequency of 350 rads/s is close to the resonant frequency of mode (1,1). It appears from Figure 9 that this mode is strongly excited with the vibration profile close to (1,1) mode shape. The displacement amplitudes along the horizontal line *y* = *b*/2 of the composite plate are presented in Figure 10. The vibration amplitude is increasing as the size of actuators increases. The vibration displacements of the composite plate predicted by Equation (12) and finite element method are in a good agreement. The maximum vibration amplitudes of the composite plate excited by the three different sizes of actuators are listed in Table 4. It shows that the difference between the present approach and finite element method is within 9%.

#### 5.1.2. Excitation Frequency *p* = *870 rads/s*

**Figure 11.**
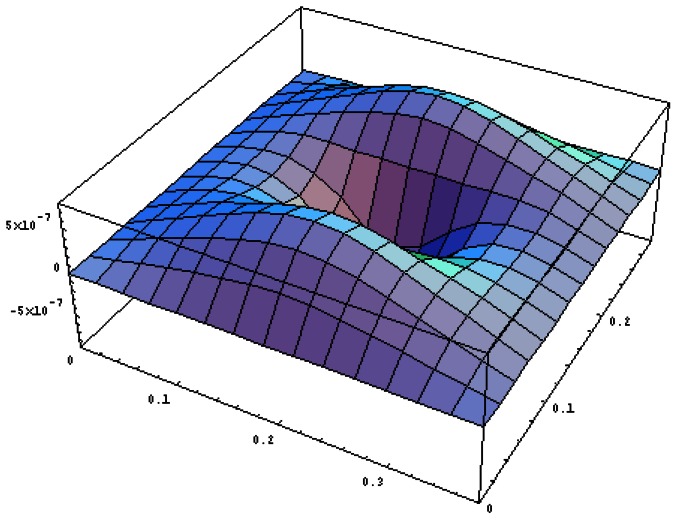
Vibration profile of the composite plate excited by PZT actuator bonded on the center of the plate with excitation frequency 870 rads/s.

**Figure 12.**
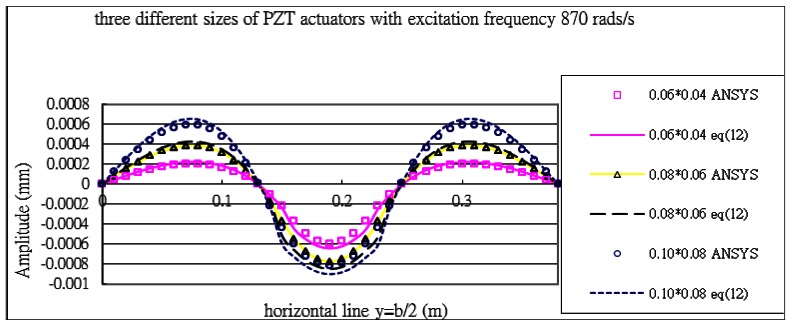
Vibration displacements of the composite plate obtained by ANSYS and Equation (12) along the horizontal line (*y* = *b*/2) excited by PZT actuator bonded on the center of the plate with excitation frequency 870 rads/s for three different sizes of actuators.

Figure 11 plots the vibration profile of the composite plate excited by the PZT patches with excitation frequency of 870 rads/s. The frequency 870 rads/s is close to the resonant frequency of mode (1,2). Similar results can be observed that this mode is strongly excited with the vibration profile close to (1,2) mode shape. The displacement amplitude distribution along the horizontal line *y* = *b*/2 in Figure 12 illustrates the evidence of the mode (1,2) being excited.

The vibration amplitude is increasing as the size of actuators increases. The vibration displacements of the composite plate predicted by Equation (12) and finite element method are in a good agreement. The maximum vibration amplitudes of the composite plate excited by the three different sizes of actuators are listed in Table 5. It shows that the difference between the present approach and finite element method is within 9%.

#### 5.1.3. Excitation Frequency *p* = *650 rads/s*

**Figure 13.**
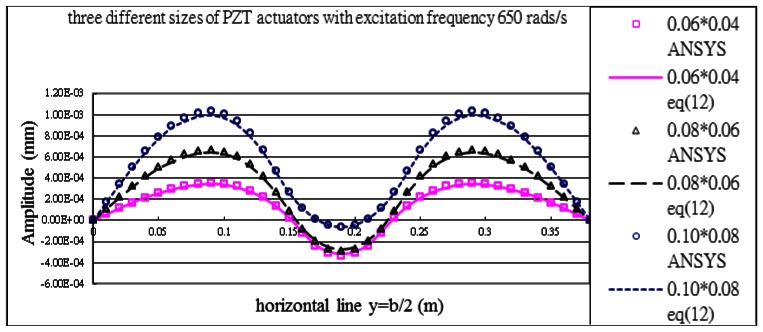
Vibration displacements of the composite plate obtained by ANSYS and Equation (12) along the horizontal line (*y* = *b*/2) excited by PZT actuator bonded on the center of the plate with excitation frequency 650 rads/s for three different sizes of actuators.

The excitation frequency was selected between modes (1,1) and (1,2), the system is being excited off the resonance. The displacement amplitude distribution along the horizontal line *y* = *b*/2 is presented in Figure 13. The vibration displacements of the composite plate predicted by Equation (12) and finite element method are in a good agreement. The maximum amplitudes of the composite plate excited by the three different sizes of actuators are listed in Table 6. It shows that the difference between the present approach and finite element method is within 5%.

### 5.2. Variation of Piezoelectric Actuator Locations

**Figure 14.**
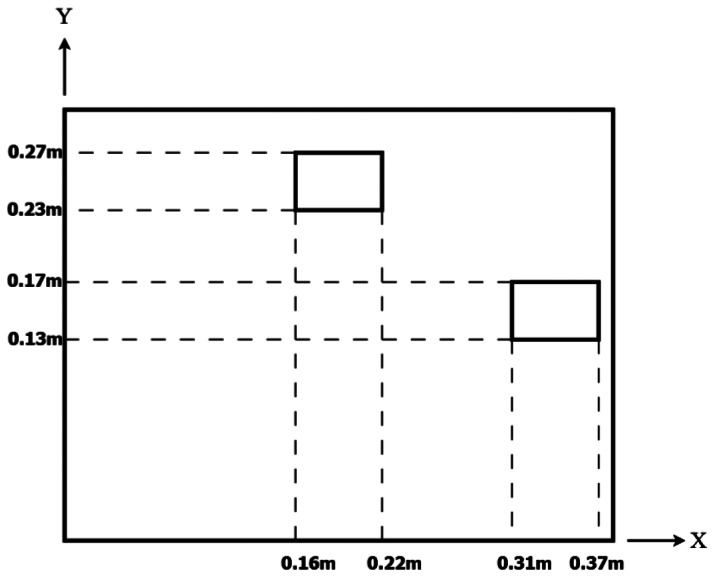
Two different locations of the PZT actuator.

In this example, the piezoelectric actuators are surface bonded at two different locations, right and top region of the plate, respectively, as shown in Figure 14. These two typical locations were arbitrarily chosen to demonstrate the influence of the actuator location on the vibration shape. The piezoelectric actuators are excited by time harmonic voltages with three different frequencies of 350, 870 and 1,050 rads/s, respectively.

#### 5.2.1. PZT Actuator Bonded on the Top Region of the Composite Plate

**Figure 15.**
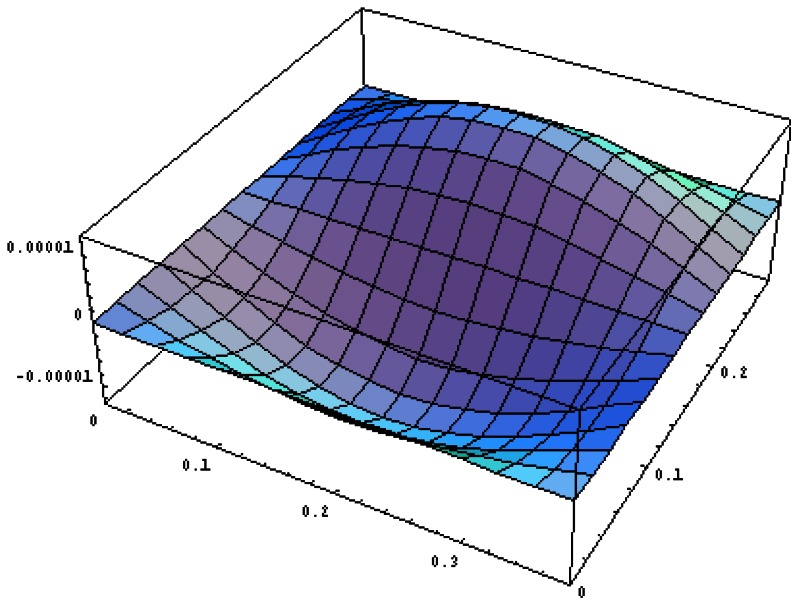
Vibration profile of the composite plate excited by PZT actuator bonded on the top region of the plate with excitation frequency 870 rads/s.

**Figure 16.**
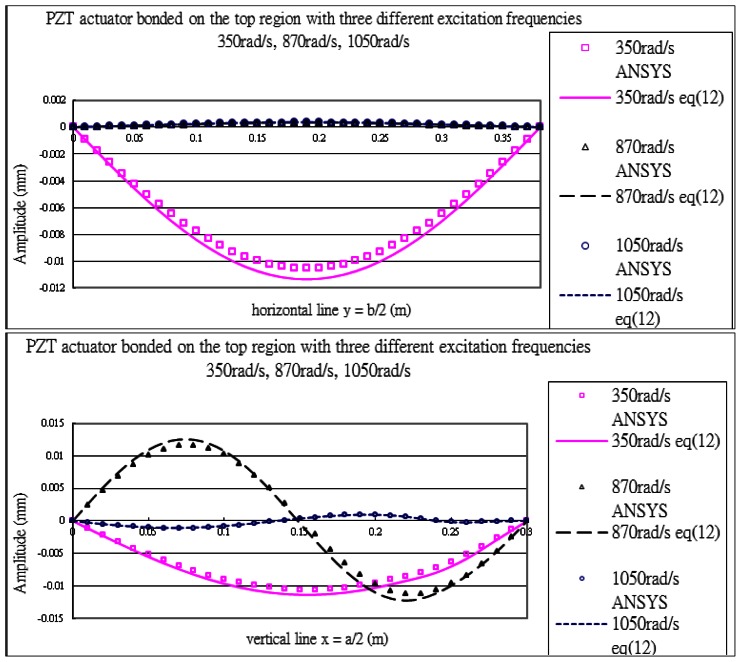
Vibration displacements of the composite plate obtained by ANSYS and Equation (12) along the horizontal line (*y* = *b*/2) and vertical line (*x* = *a*/2) excited by PZT actuator bonded on the top of the plate for three different excitation frequencies.

The vibration profile of the composite plate excited by the PZT actuators surface bonded on the top region of the plate with excitation frequency 870 rads/s are shown in Figure 15. The displacement amplitudes of the plate along the horizontal line *y* = *b*/2 and vertical line *x* = *a*/2 are presented in Figure 16. The vibration displacements of the plate obtained by the present approach of Equation (12) and finite element method are in close agreement. Table 7 lists the maximum amplitudes of the plate induced by the piezoelectric actuators surface bonding on the top location. It shows that the difference between the present approach and finite element method is within 8%.

#### 5.2.2. PZT Actuator Bonded on the Right Region of the Composite Plate

**Figure 17.**
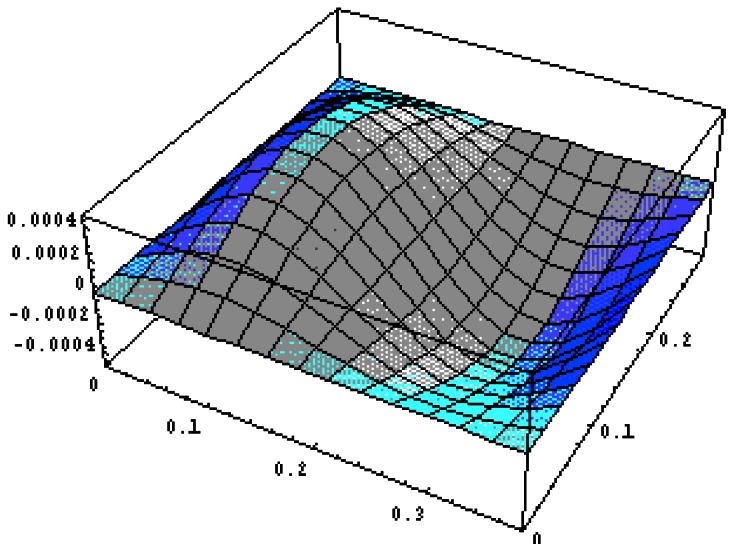
Vibration profile of the composite plate induced by PZT actuator bonded on the right region of the plate with excitation frequency 1,050 rads/s.

**Figure 18.**
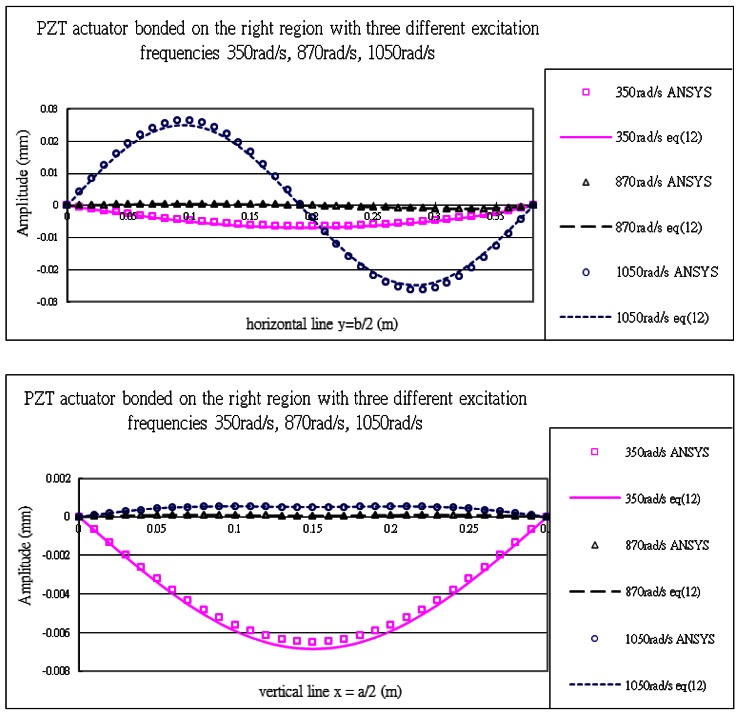
Vibration displacements of the composite plate obtained by ANSYS and Equation (12) along the horizontal line (*y* = *b*/2) and vertical line (*x* = *a*/2) excited by PZT actuator bonded on the right region of the plate for three different excitation frequencies.

The vibration profile of the composite plate excited by the PZT actuators surface bonded on the right region of the plate with excitation frequency 1,050 rads/s are shown in Figure 17. The displacement amplitudes of the plate along the horizontal line *y* = *b*/2 and vertical line *x* = *a*/2 are presented in Figure 18. The vibration displacements of the plate obtained by the present approach of Equation (12) and finite element method are in close agreement.

Table 8 lists the maximum amplitudes of the plate induced by the piezoelectric actuators surface bonding on the right region. It shows that the difference between the present approach and finite element method is within 7%. The difference of the vibrating shapes shown in Figures 9, 15 and 17 demonstrates that the vibration of the plate can be controlled by placing the actuators at various locations.

## 6. Conclusions

A model for the excitation of a composite laminate plate by patch type piezoelectric actuators surface bonded to the structure has been developed. The model has been applied to a simply supported cross-ply composite laminate plate excited by two piezoelectric actuators symmetrically bonded to both sides of the plate with time harmonic electric loading. An analytical expression of the harmonic vibration of the simply supported composite laminate induced by the piezoelectric actuators is presented. Numerical examples demonstrate that it is possible to excite the vibration modes by patch type actuators. When the input frequency is close to the resonant frequency of a mode, that mode can be excited provided the actuator is properly located. Three-dimensional finite element analysis was conducted using the commercial software ANSYS, and compared with the theoretical prediction. Good agreement was observed between the numerical result and theoretical prediction. Parametric study shows that the input frequency and location significantly affect the ability of the piezoelectric actuators to excite certain modes. Present study demonstrates the potential of controlling vibration in a plate structure using two dimensional patch type actuators. The optimal placement and configuration of PZT actuators will be the subject of future work.

## Figures and Tables

**Table 1.** Material properties of carbon/epoxy.

**Longitudinal Modulus *E*_1_**	**Transverse Modulus *E*_2_**	**Shear Modulus *G*_12_**	**Shear Modulus *G*_23_**	**Poisson's Ratio *v*_12_**	**Poisson's Ratio *v*_23_**
108 GPa	10.3 GPa	7.13 GPa	4.02 GPa	0.28	0.28

**Table 2.** Natural frequencies (rad/s) of the composite plate obtained by finite element method.

		**n**		

		**1**	**2**	**3**
m	1	358.5	875.3	1805.3
	2	1056.4	1433.6	2247.3
	3	2262.8	2561.8	3223.7

**Table 3.** Natural frequencies (rad/s) of the composite plate obtained by theoretical prediction Equation (10).

		n		

		1	2	3
m	1	359.1	876.3	1806.9
	2	1057.6	1436.2	2251.4
	3	2266.1	2566.8	3231.6

**Table 4.** Maximum vibration amplitudes (mm) excited by three different sizes of PZT actuator bonded on the center of the plate with excitation frequency 350 rads/s.

**PZT Size**	**Finite Element ANSYS**	**Theoretical Prediction** Equation (12)	**Difference %**
			
0.06 m × 0.04 m	3.53 × 10^−2^	3.33 × 10^−2^	6.0
0.08 m × 0.06 m	1.66 × 10^−1^	1.56 × 10^−1^	6.4
0.10 m × 0.08 m	1.15	1.06	8.5

**Table 5.** Maximum vibration amplitudes (mm) excited by three different sizes of PZT actuator bonded on the center of the plate with excitation frequency 870 rads/s.

**PZT Size**	**Finite Element ANSYS**	**Theoretical Prediction** Equation (12)	**Difference %**
0.06 m × 0.04 m	5.95 × 10^−4^	6.42 × 10^−4^	7.4
0.08 m × 0.06 m	7.74 × 10^−4^	8.49 × 10^−4^	8.8
0.10 m × 0.08 m	1.17 × 10^−3^	1.12 × 10^−3^	4.3

**Table 6.** Maximum vibration amplitude (mm) excited by three different sizes of PZT actuator bonded on the center of the plate with excitation frequency 650 rads/s.

**PZT Size**	**Finite Element ANSYS**	**Theoretical Prediction** Equation (12)	**Difference %**
0.06 m × 0.04 m	0.516 × 10^−3^	0.508 × 10^−^3	1.5
0.08 m × 0.06 m	0.100 × 10^−2^	0.980 × 10^−2^	2.0
0.10 m × 0.08 m	0.158 × 10^−2^	0.151 × 10^−2^	4.7

**Table 7.** Maximum vibration amplitudes (mm) excited by PZT actuator bonded on the top of the composite plate for three different excitation frequencies.

**Frequency rads/s**	**Finite Element ANSYS**	**Theoretical Prediction** Equation (12)	**Difference %**
350	1.05 × 10^−2^	1.13 × 10^−2^	7.4
870	1.16 × 10^−2^	1.25 × 10^−2^	7.3
1050	1.11 × 10^−3^	1.05 × 10^−3^	4.8

**Table 8.** Maximum vibration amplitudes (mm) excited by PZT actuator bonded on the right region of the composite plate for three different excitation frequencies.

**Frequency rads/s**	**Finite Element ANSYS**	**Theoretical Prediction** Equation (12)	**Difference %**
350	6.48 × 10^−3^	6.84 × 10^−3^	5.2
870	1.09 × 10^−3^	1.01 × 10^−3^	7.0
1050	2.64 × 10^−3^	2.49 × 10^−2^	6.0
